# Intensification of Biodiesel Processing from Waste Cooking Oil, Exploiting Cooperative Microbubble and Bifunctional Metallic Heterogeneous Catalysis

**DOI:** 10.3390/bioengineering9100533

**Published:** 2022-10-08

**Authors:** Fahed Javed, Muhammad Rizwan, Maryam Asif, Shahzad Ali, Rabya Aslam, Muhammad Sarfraz Akram, William B Zimmerman, Fahad Rehman

**Affiliations:** 1 Microfluidics Research Group, Department of Chemical Engineering, COMSATS University Islamabad, Lahore 54000, Pakistan; 2 Institute of Energy and Environmental Engineering, University of Punjab, Lahore 54000, Pakistan; 3 Institute of Chemical Engineering and Technology, University of Punjab, Lahore 54000, Pakistan; 4 Department of Chemical & Biological Engineering, The University of Sheffield, Sheffield S1 3JD, UK

**Keywords:** biodiesel, waste cooking oil, transesterification, bifunctional catalyst, microbubble technology

## Abstract

Waste resources are an attractive option for economical the production of biodiesel; however, oil derived from waste resource contains free fatty acids (FFA). The concentration of FFAs must be reduced to below 1 wt.% before it can be converted to biodiesel using transesterification. FFAs are converted to fatty acid methyl esters (FAMEs) using acid catalysis, which is the rate-limiting reaction (~4000 times slower than transesterification), with a low conversion as well, in the over biodiesel production process. The study is focused on synthesizing and using a bifunctional catalyst (7% Sr/ZrO_2_) to carry out esterification and transesterification simultaneously to convert waste cooking oil (WCO) into biodiesel using microbubble-mediated mass transfer technology. The results reveal that a higher conversion of 85% is achieved in 20 min using 7% Sr/ZrO_2_ for biodiesel production. A comprehensive kinetic model is developed for the conversion of WCO in the presence of a 7% Sr/ZrO_2_ catalyst. The model indicates that the current reaction is pseudo-first-order, controlled by the vapor–liquid interface, which also indicates the complex role of microbubble interfaces due to the presence of the bifunctional catalyst. The catalyst could be recycled seven times, indicating its high stability during biodiesel production. The heterogeneous bifunctional catalyst is integrated with microbubble-mediated mass transfer technology for the first time. The results are unprecedented; furthermore, this study might be the first to use microbubble interfaces to “host” bifunctional metallic catalysts. The resulting one-step process of esterification and transesterification makes the process less energy-intensive and more cost-efficient, while also reducing process complexity.

## 1. Introduction

The current standard of living is substantially dependent on energy. Its generation is a measure of progress for a developing country. Energy is consumed in domestic and industrial sectors generated primarily by fossil fuels and other sources. Biodiesel production is considered a potential alternative to fossil fuels due to its numerous advantages, such as nontoxicity and biodegradability, with low harmful emissions during combustion. Generally, biodiesel is produced via two routes: (1) esterification reaction of free fatty acids (FFA), and (2) transesterification of triglycerides, both with alcohol [1,2]. Homogeneous catalysts such as NaOH, KOH, H_3_PO_4_, H_2_SO_4_, and HCL are usually preferred due to their higher degree of interaction because of superior miscibility [3,4]. However, homogenous catalysts dissolved in the reaction mixture cause numerous challenges during the downstream separation and purification stages [5,6]. Biodiesel production through heterogeneous catalysts is an advantageous alternative to homogeneous catalysts as it provides numerous advantages, including reduced unit operation requirement, non-corrosiveness, low waste generated, reusability, recyclability, and ease of product separation [7,8]. Basic catalysts are generally used for converting triglycerides into biodiesel, but they also saponify FFAs in the feedstock, resulting in emulsions that add steps for purification, while reducing yield [9,10]. Acidic catalysts are generally used to convert FFA through esterification and transform triglycerides via hydrolysis into diglycerides, which can further convert them into FFA and cause catalyst deactivation through leaching [11,12]. To overcome the drawbacks related to the individual acidic and basic heterogeneous catalysts, bifunctional catalysts were introduced. Bifunctional catalysts can combine the characteristics of both acidic catalysts that can transform FFAs and basic catalysts that tackle triglycerides in the feedstock [13,14]. Furthermore, saponification generated by FFAs and water can be completely avoided using a bifunctional catalyst. An effective bifunctional catalyst with amphoteric base material on which acidic or basic promotors can further be modified is required. Zirconium oxide (ZrO_2_) is amphoteric in nature. It has been reported to be an efficient base for heterogeneous catalysts due to its mechanical strength, corrosion resistance, chemical stability, and high water retention [6,15]. Recently, ZrO_2_ has been modified to yield acidic forms, i.e., tungstate zirconia alumina (Al_2_O_3_/ZrO_2_, TiO_2_/ZrO_2_) and basic forms such as CaO, and La_2_O_3_ [16,17]. Thus, ZrO_2_ can be modified and improved to design specific catalysts with desired properties.

The use of solid heterogeneous catalysts enhances the ability of biodiesel production from WCO without additional treatment [18,19]. However, a long reaction time is still a major challenge in biodiesel production. Jitputti et al. investigated the comparison of zirconia and sulfated zirconia and achieved conversion of 49.3% and 86.3% of crude palm kernel oil, respectively, in almost 4 h [20]. Jamil et al. investigated the Mn@MgOZrO_2_ bifunctional catalyst for waste *Phoenix dactylifera* L. oil and achieved 96.4% biodiesel conversion in 4 h [21]. Current results collectively indicate that bifunctional catalysts have enhanced performance, but an efficient process method is needed that reduces reaction time and increases the reaction rate [22,23]. 

Microbubble technology was recently introduced for biodiesel production, yielding improved results by enhancing the mass transfer and rate of reaction via alcohol injection within the microbubble phase [24,25]. Microbubbles have less buoyancy force, high surface energy, high temperature, and high residence time due to their smaller size than macrobubbles. When the bubble rises in the laminar regime due to its smaller size and provides internal mixing at the bubble and oil interface, homogeneity is achieved within a millisecond. Microbubble formation at a low flow rate is more favorable for smaller bubble formation, reduced coalescence, and higher surface energy as a result mass transfer increases, which directly increase the conversion of the process [2,26]. Ahmed et al. reported an increase in the reaction rate and reduced reaction time of oleic acid and methanol (MeOH) using microbubbles and achieved approximately 96% conversion of biodiesel in just 0.5 h [27]. Javed et al. studied acid esterification using chicken fat oil and MeOH and achieved 89% conversion in 0.5 h [28]. These results illustrate that microbubble technology promises an increasing rate of reaction by converting the liquid–liquid bulk reaction into a gas–liquid interfacial reaction. All these studies provide sufficient evidence that the reaction occurs at the MeOH/oil interface. However, the current work emphasizes another amphoteric characteristic (bifunctional catalyst) present on the bubble interface. The competition of microbubbles and particles at interfaces is a well-known industrial process due to dissolved air flotation, which is also intensified by fluidic oscillated microbubbles [29,30]. The current study is based on synthesizing a heterogeneous bifunctional catalyst and integrating it with microbubble-mediated mass transfer technology to enhance the reaction rate and overall process conversion. To the best of the authors’ knowledge, this is the first work that uses microbubble interfaces to “host” bifunctional metallic catalysts. The closest work used bimetallic catalysts to enhance the hydroxyl radical production from ozone microbubbles, but illustrated the mechanism as bubble–pellet transient collisions, where the pellets are much larger than the microbubbles [31]. There is only one similar study for single-metal catalysis, which implemented ozone microbubble dissociation into hydroxyl radicals for oxidation reactions [32]. Here, the traditional two-step bio-diesel production process was converted into a single-step process by carrying out esterification and transesterification simultaneously, making the process more energy- and cost-efficient, while also reducing process complexity. It should be noted that esterification reactions are, in general, equilibrium reactions that achieve 60–80% conversion without reactive separation. The high conversions demonstrated in this paper give credence to the suggestion that the microbubble interface, populated by bimetallic catalyst particles, serves as the heterogeneous catalyst interface, while the vapor-phase product (water) is simultaneously extracted. 

In the current study, efforts are made to present a sustainable approach of generating energy in the form of biodiesel from waste. Strontium zirconium oxide (7% Sr/ZrO_2_) was synthesized and integrated with rapidly developing microbubble technology from WCO. The synthesized heterogeneous catalyst was characterized using various analytical techniques. To increase its applicability on a commercial scale and to optimize the process, additional parameters were studied. Furthermore, a detailed analysis of the reaction kinetics, catalytic mechanism, and process economics was also conducted. Hence, this study can contribute additional insight into the integration of heterogeneous catalysts with acidic and basic active sites using microbubble technology to produce high-quality biodiesel.

## 2. Materials and Methods

### 2.1. Materials 

Analytical-grade FAMEs, hexane, zirconium(IV) butoxide, strontium nitrate, and poly(ethylene glycol)-block-poly(propylene glycol)-block-poly(ethylene glycol) ((EO)20(PO)70(EO)20) triblock copolymer (TBC) were purchased from Sigma Aldrich. Analytical-grade MeOH (99%) and butanol were procured from DAEJUNG chemicals, Siheung-si, South Korea. WCO was collected from the university cafeteria in Lahore, Pakistan. All other chemicals used for catalyst preparation were purchased from Sigma Aldrich, St. Louis, MO, USA.

### 2.2. Oil Purification

WCO was filtered and then washed five times with distilled water at 60 °C until neutral pH was achieved. Afterward, WCO was dried over an anhydrous sodium sulfate bed to remove traces of water. The dried WCO was then stored in a sealed bottle, and its composition was characterized using GC–MS analysis. The properties of WCO and its oil composition are presented in Table 1.

### 2.3. Catalyst Preparation

Zirconium(IV) butoxide and strontium nitrate (Sr(NO_3_)_2_) were used as the precursors to synthesize the mesoporous specimen. Approximately 5.0 g of TBC was dissolved in 50.0 mL of ethanol and left to stir for 4 h at room temperature. Then, 80 mmol of zirconium(IV) n-butoxide (80 wt.% solution in 1-butanol) was dissolved in 20 mL of 68–70 wt.% nitric acid and 50.0 mL of ethanol. Once dissolved, a calculated amount of strontium metal solution (1.0 M) was added to a flask and stirred for 2 h. The pH was carefully maintained at 12 using 2 M NaOH solution. The solution was heated under continuous slow stirring at room temperature for 4 h. Subsequently, the two solutions were combined, and 30.0 mL of distilled water for complete transfer of the solutions. The combined solution was stirred for 5 h at room temperature. The solvent was removed at 100 °C for 24 h in the oven. Lastly, the catalyst was calcined under air in the furnace at 550 °C for 5 h. The maximum conversion of biodiesel was achieved using 7% Sr loading during the preliminary experiments, due to which 7% Sr was used throughout the study to optimize the amount to impregnate ZrO_2_.

### 2.4. Characterization of Catalyst

Fourier-transform infrared spectroscopy (FTIR) analysis with a Thermo-Nicolet 6700P Spectrometer, Thermo Fisher Scientific, Waltham, MA, USA, was used to detect the functional groups of 7% Sr/ZrO_2_. The wavelength of FTIR was set in the range of 800 to 4000 cm^−1^. To study the surface morphology of the catalyst, scanning electron microscopy (SEM) (FEI Nova 450 NanoSEM, Thermo Fisher Scientific, Waltham MA, USA,) was used. To identify the effect of strontium on the crystallinity of ZrO_2_, X-ray diffraction (XRD) was used. For XRD (Equinox 2000, Thermo Fisher Scientific, USA), the range of 2θ = 2°–116° was selected using Cu-Kα radiation (λ = 0.145 nm). For surface area and porosity analysis, a Micromeritics TriStar II-3020 analyzer (Micromeritics, Norcross, GA, USA) was used to obtain N_2_ adsorption/desorption isotherms of the catalyst at 77.3 K. The Brunauer–Emmett–Teller (BET) adsorption approach was used to infer the surface area. The pore surface area and volume were determined using the t-plot method. 

### 2.5. Experimental Procedure

Biodiesel was produced through both esterification and transesterification, during which WCO reacted with MeOH in the presence of 7% Sr/ZrO_2_. Initially, MeOH was heated using a heating mantle around its boiling point in a round-bottom flask. Meanwhile, WCO and 7% Sr/ZrO_2_ were premixed in a separate beaker for a specific time interval. The pre-mixed solution was then transferred to the microbubble reactor. The microbubble reactor consisted of sintered-borosilicate diffuser (40 to 16 µm pore size) with a total reactor volume of 500 mL. MeOH was injected from the bottom of the reactor in the form of vapor. The temperature of the reactor was maintained at 70 °C using a brisk heater. The reaction was terminated when the desired quantity of MeOH passed through the reactor.

**Figure 1:**
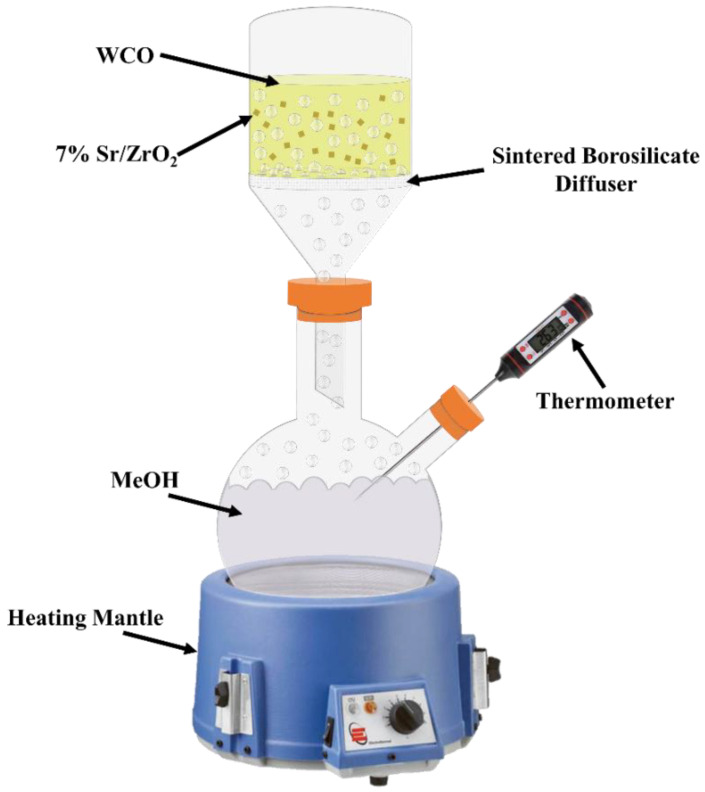
Systematic diagram of microbubble technology for biodiesel production.

Afterward, biodiesel samples were centrifuged at 3500 rpm for 10 min to separate the catalyst from biodiesel, and the samples were washed with deionized water to remove impurities. For complete water removal, samples were dried using a rotary evaporator (Buchi R-210, BUCHI Corporation, New Castle, DE, USA). For parametric study, the molar ratio of WCO and MeOH was varied from 1:5 to 1:25. Catalyst loading ranged from 0 to 3 wt.% WCO. The temperature was varied from 70 to 90 °C to study the effect of biodiesel production and activation energy. All experiments were carried out three times to calculate the standard error. A diagram of the process is given in Figure 1.

### 2.6. Biodiesel Analysis

The analysis process of biodiesel using GC was taken from the literature [27,28]. Briefly, a Shimadzu GC-2014, Shimadzu Europa, Duisburg, Germany, was used to analyze the biodiesel with a GC-FID and column Agilent J&W EN14103 Column, Agilent Technologies, Santa Clara, CA, USA, with 30 m length, 0.32 mm id, and 0.25 µm film thickness. Nitrogen gas with a flowrate of 1.5 mL/min was used as the carrier gas. The conditions were kept constant for every sample to achieve comparable readings. The sample was injected at 523 K and a split ratio of 50:1 [27,28].

## 3. Results and Discussion

### 3.1. Catalyst Analysis

**Figure 2:**
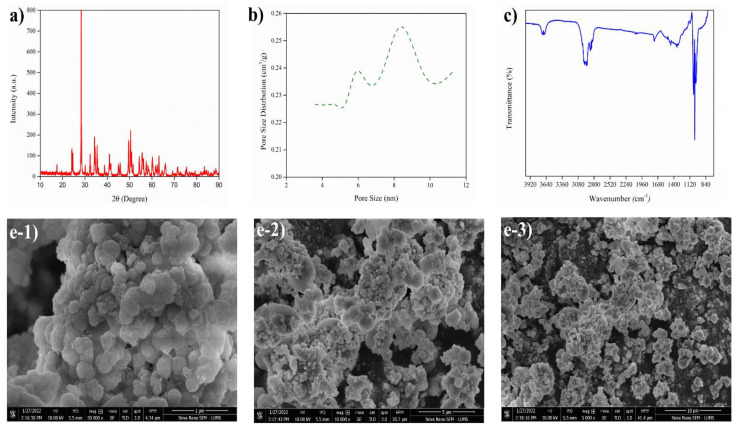
Characterization of Sr/ZnO_2_: (**a**) XRD analysis, (**b**) BET analysis, (**c**) FTIR, and (**e-1**–**3**) SEM analysis.

The characterization of 7% Sr/ZrO_2_ is shown in Figure 2a–c,e. The FTIR spectrum of zirconia shows a broad peak band in the region of 3700–3400 cm^−1^, which was attributed to asymmetric stretching of –OH groups. The band at 900 cm^−1^ was associated with ZrO. The peak at 1623 cm^−1^ corresponded to the C=O group due to SrO_2_ in the catalyst. The weak absorption bands at 1603 cm^−1^ and 1318 cm^−1^ were attributed to the bending vibration of C–H bands. Pore size and surface area analyses showed that the catalyst formation was mesoporous, ranging from 3 to 11 nm. The XRD diffractograms of synthesized Sr/ZrO_2_ revealed the crystal structure of pure ZrO_2_ to be monoclinic. The structure of ZrO_2_ was sustained after calcination at 550 °C for 5 h, with no peak broadening observed. The pattern of Sr/ZrO_2_ predominantly showed peaks of ZrO_2_ as the parent peak, with an additional peak at 31.750° for Sr/ZrO_2_. The pore size distribution of the catalyst is shown in Figure 2b, and the pore size and surface area data estimated via BET analysis are shown in Table 2. The catalyst pore size demonstrated mesopores ranging between 3 and 11 nm. 

**Figure 3:**
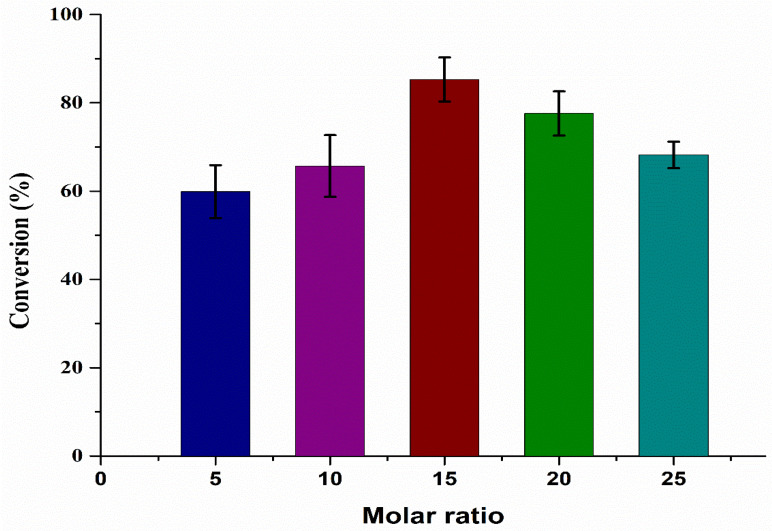
Effect of molar ratio (WCO/MeOH = 1:5 to 1:25) on biodiesel production.

Figure 2e-1–3 show the 7% Sr/ZrO_2_ surface morphology at different magnitudes using SEM. The modified ZrO_2_ showed well-shaped crystalline particles after impregnation of Sr particles. The analysis revealed that the addition of Sr metal to ZrO_2_ increased the amphoteric behavior of ZrO_2_, which increased both the basic and the acidic active sites of the catalyst. The presence of both acidic and basic sites in 7% Sr/ZrO_2_ facilitated both esterification and transesterification. 

### 3.2. Parameter Optimization for Biodiesel Production

#### 3.2.1. Effect of Molar Ratio on Biodiesel Production

To study the effect of molar ratio on WCO and MeOH for biodiesel production, different experiments were performed by changing the molar ratio (WCO/MeOH = 1:5 to 1:25), as shown in Figure 3. Initially, the results indicated that, by increasing the molar ratio from 1:5 to 1:15, the reaction conversion increased from 59% to 85%, before decreasing to 68% at a higher molar ratio of 1:25. Increasing the molar ratio also increased the process conversion. By increasing the MeOH quantity, the reaction time of the system also increased, due to which the reaction moved further in the forward direction. Furthermore, the system operated at a temperature higher than the boiling point of MeOH, which resulted in unreacted MeOH leaving via the top of reactor. However, the MeOH volume could be increased by changing the molar ratio to an extent that a limited amount of MeOH stayed in the reactor, as reported by Javed et al. (2021) [28]. The presence of unreacted MeOH in the system diminished the active catalyst sites due to oil dilution, which reduced the catalyst activity [33,34]. Hence, the process efficiency decreased at higher molar ratios, as observed from the current result. The optimal molar ratio was found to be 1:15.

#### 3.2.2. Influence of Catalyst on the Conversion of WCO

**Figure 4:**
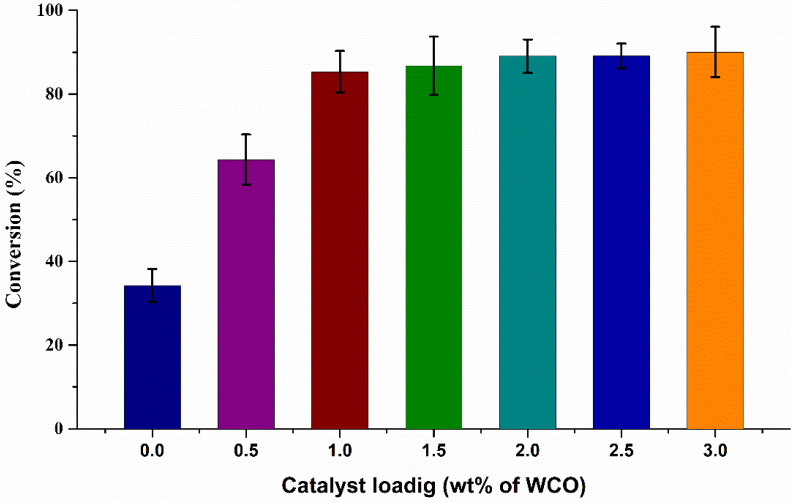
Influence of catalyst loading on the conversion of WCO.

The effect of catalytic activity using 7% Sr/ZrO_2_ is shown in Figure 4. The microbubble process yielded a low conversion of 34% in the absence of catalyst during the transesterification reaction. The conversion of the transesterification reaction was related to the active sites of the 7% Sr/ZrO_2_ catalyst. The conversion of the process increased from 64% to 90% upon increasing catalyst loading. Increasing the catalyst loading seemingly enhanced the entanglement of WCO and 7% Sr/ZrO_2_ catalyst. The degree of entanglement of WCO and 7% Sr/ZrO_2_ affected the conversion of WCO, whereby a higher degree of entanglement led to a higher conversion of WCO and a higher rate of reaction, and vice versa. However, the results illustrate that, beyond 1% catalyst loading, only a 5% increase in conversion was obtained upon doubling catalyst loading. One possible reason is that increasing the catalyst loading increased the number of active sites, whereas the reactive side of WCO remained constant during entanglement, such that excess loading of 7% Sr/ZrO_2_ did not drastically enhance the conversion of the transesterification reaction. Furthermore, the results demonstrate that 1% catalyst loading was optimal for the current study.

#### 3.2.3. Effect of Temperature on Biodiesel Production

**Figure 5:**
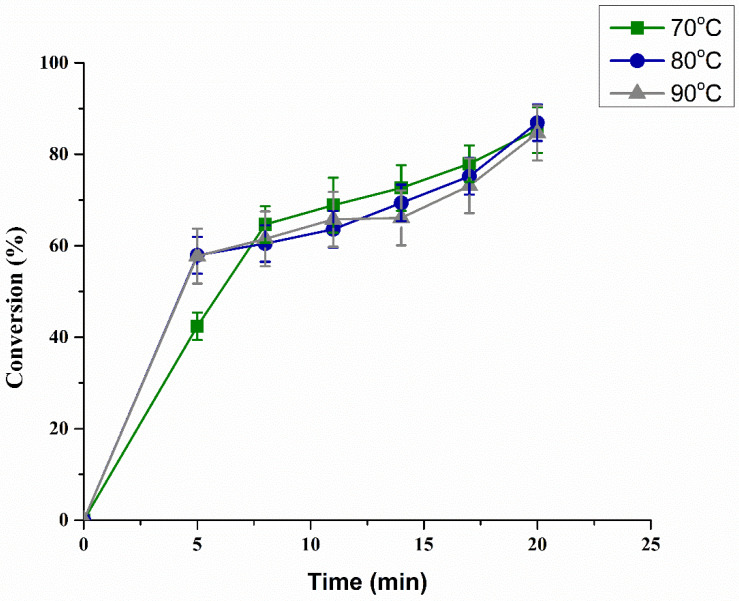
Effect of different temperatures on WCO conversion into biodiesel.

The effect of temperature is a vital parameter controlling the reaction rate of the process. However, raising the temperature also increases the processing costs and renders the process unviable for commercial scale. The temperature was varied from 70 to 90 °C to study the impact of temperature on the process (Figure 5). The results indicate that the conversion of WCO was not significantly affected by the change in temperature, as the initial temperature of the reactor was above the boiling point of MeOH. When bubbles rose, they reacted with WCO, while unreacted MeOH left the system, thereby not affecting the overall conversion of the process. However, at the start of the reaction, the high-temperature system showed a higher conversion of 58% in 5 min at 90 °C than that achieved at 70 °C (42%). A possible explanation for this behavior is that, at high temperature, the viscosity of oil decreased, due to which the miscibility of 7% Sr/ZrO_2_ and MeOH increased with WCO. Furthermore, by increasing the temperature, the collision frequency of bubbles with WCO also increased, which also enhanced the rate of reaction. Hence, the optimal and most economical temperature for this study was 70 °C.

#### 3.2.4. Effect of Reaction Time on Biodiesel Production

**Figure 6:**
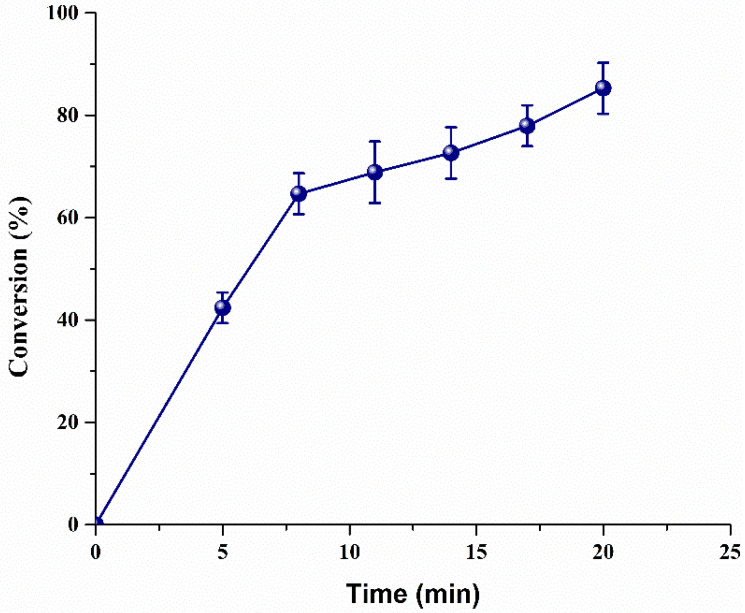
Biodiesel production over time using microbubble technology.

The biodiesel production was monitored in a reaction using WCO and MeOH as the model system. Before measuring biodiesel production using heterogeneous catalysts in the microbubble system, it was necessary to perform a control experiment using 7% Sr/ZrO_2_ under the same conditions by mimicking the conventional process with a beaker and magnetic stirring. For both processes, 100 g of WCO, 72 mL of MeOH, and 1 wt.% of 7% Sr/ZrO_2_ were used. Only 8% conversion was achieved in 20 min, eventually reaching 93% in 240 min. However, in the current microbubble system, MeOH was introduced in the microbubble phase, enhancing the diffusion rate of both reactants, in addition to increasing the system mass transfer efficiency [27,28]. As indicated from the microbubble results (Figure 6**),** a higher conversion of 85% was achieved in 20 min.

The first 8 min of the reaction occurred spontaneously, as illustrated from the steep curve, achieving 65% conversion, before gradually increasing to 85% in 20 min. The possible reason for this is that, at the start of the reaction, the WCO and catalyst had readily available active sites for reaction as time increased, and the concentration of WCO was reduced by more than 65% via conversion into biodiesel; accordingly, bubbles had a lower chance of interaction with the freshly available biodiesel. Consequently, the rate of reaction slowed down during the last 12 min. A comparison of the study with other heterogeneous catalysts is shown in Table 3. The current study clearly achieved a higher reaction rate in a shorter period than previously reported studies.

**Table 3:** Comparison of the current study with other heterogeneous catalysts used for biodiesel production.

Catalyst	Temperature (°C)	Time (min)	Catalyst Loading (wt.%)	Conversion (%)	Reference
Li-Al HTA	65	60	3	83	[35]
KI/SiO	70	480	5	91	[36]
KF/Al_2_O_3_	60	480	3	90	[37]
3% La_2_O_3_–ZrO_2_	65	300	6	56	[38]
ZrO_2_/SiO_2_	120	120	10	48.6	[6]
Li/ZrO_2_	65	180	3	98.2	[39]
21% La_2_O_3_/ZrO_2_	200	480	5	84.9	[40]
7% Sr/ZrO_2_	70	20	1	85	This study

### 3.3. Reaction Kinetics and Mechanism of WCO-Based Biodiesel

#### 3.3.1. Proposal of a Reaction Mechanism for Biodiesel Production Using 7% SR/ZRO_2_

**Figure 7:**
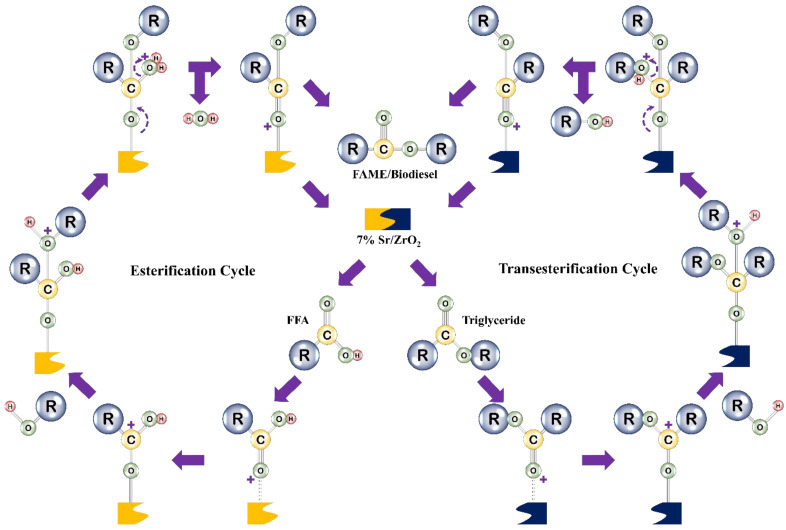
Proposed reaction of 7% Sr/ZrO_2_ for converting WCO into biodiesel.

The current study proposes that 7% Sr/ZrO_2_ performed both esterification and transesterification simultaneously to produce biodiesel. ZrO_2_ is amphoteric and possesses both basic and acidic active sites. The active side was further strengthened by modifying it with Sr, which further improved the catalyst activity. The proposed reaction mechanism for this study is shown in Figure 7, which further elaborates how esterification and transesterification occurred on the acidic and basic active sites of the catalyst. The chemical reaction proposed through the heterogeneous catalyst is based on three basic steps: adsorption of the reactant on active sites of the catalyst, reaction between active sites of the catalyst, and desorption of product from active sites of the catalyst.

In biodiesel production, both FFA and MeOH molecules get absorbed onto the acidic and basic catalyst sites. The FFA molecules change to carbocations, with oxygen anions from MeOH. In the second step, a nucleophilic attack of the carbocation and oxygen anion is directed toward the molecules of triglycerides and FFA, thereby supporting both esterification and transesterification. Furthermore, tetrahedral intermediates are also formed due to nucleophilic attacks. In the last step, –OH and –C–O– bonds break. As a result, hydroxyl group and alkyl triglycerides are desorbed. Desorption of these molecules provides us with a final product known as biodiesel (mono-alkyl ester). After desorption of biodiesel, both acidic and basic sites of the catalyst are again available for another cycle. This process continues until the reaction is completed; moreover, water and glycerol are produced as byproducts of this process.

#### 3.3.2. Kinetics Analysis and Activation Energy of WCO-Based Biodiesel

To investigate the reaction kinetics of the vapor–liquid system using heterogeneous catalysts, different parameters were optimized in the above experiments, and the kinetics were determined in optimal conditions. To validate the current hypothesis of the catalyst facilitating the reaction on the bubble surface while moving the bubble upward, the Hatta number (*Ha*) was calculated using Equation (1) [41,42]. Ha signifies whether the reaction occurred on the surface of the bubble or in bulk. If the value of *Ha* is greater than 1, then the reaction occurred on the surface of the bubble, and the controlling factor was the reaction kinetics. If the reaction occurred in bulk, then mass transfer was the controlling factor, with intense mixing becoming the dominant factor.

(1) $$Ha=\frac{\sqrt{{\left(D_{o}\right)}_{T}\;k\;C_{b}}}{k_{bl}},$$

where *k* is the rate constant, *k_bl_* is a liquid film coefficient, and *D_o_* is a coefficient of diffusion of MeOH in WCO at different temperatures (see Equations (2) and (3) [43], where *v_l_* and *v_g_* are molar volumes of WCO and MeOH, and *µ_l_* is the viscosity of WCO).

(2) $${\left(D_{o}\right)}_{25{}^{\circ}C}=6.02\times 10^{-5}\left(\frac{V_{l}^{0.36}}{\mu_{l}^{0.61}V_{g}^{0.64}}\right).$$

(3) $${\left(D_{o}\right)}_{T}=4.996\times 10^{3}{\left(D_{o}\right)}_{25{}^{\circ}C}\exp\left(\frac{-2539}{T}\right).$$

The value of *k_bl_* was calculated using Equation (4) for bubble sizes less than 2 mm [44].

(4) kbl=0.31((Dg/l)2ρlgμl)13.

In the current study, a calculated value of *Ha* > 1 implied that the reaction occurred on the surface of the bubble and that the catalyst active site was induced by the MeOH bubble. To calculate the order of reaction of the vapor–liquid system, the enhancement factor (*E*) was determined using Equation (5) [41], where *E_i_* is the infinite enhancement factor, calculated using Equation (6).

(5) $$\;E=Ha\left(1-\frac{Ha-1}{2E_{i}}\right).$$

(6) $$E_{i}=1+{\left(D_{o}\right)}_{T}\left(\frac{C_{l}H}{b\;P_{g}}\right),$$

where *C_l_* is the WCO concentration (kmol·m^−3^), *P_g_* is the partial pressure of vapors, and *H* is Henry’s constant. The values of E and Ha indicate that the reaction is pseudo-first-order due to the similarity of both values. The rate was determined using Equation (7).

(7) $$-r_{A}=\frac{1}{\frac{1}{k_{a}\sigma }+\frac{H}{a\sqrt{{\left(D_{o}\right)}_{T}kC_{b}}}}P_{a},$$

where *k_a_* is the gas film coefficient, and *P_a_* (bar) is the pressure of the system. The calculated values of *k_a_* with the interfacial area (*k_a_* *σ*), *k_bl_*, and H were 0.011 kmol·s^−1^·m^−3^, 1.04 × 10^−4^ ms^−1^, and 3.72 kmol·m^−3^·Pa^−1^, respectively. The overall rate of reaction is shown in Equation (8).

(8) $$-r_{A}=\left(8.730\times 10^{-5}\right)\left(P_{a}\times 101325\right)\left(\sqrt{C_{b}}\right).$$

The current kinetics show that the order of the reaction was pseudo-first-order, and that the reaction occurred on the microbubble surface, with the concentration gradient of WCO and MeOH remaining high throughout the reaction. The high concentration gradient and high surface area of catalyst facilitated biodiesel production in a shorter time.

**Figure 8:**
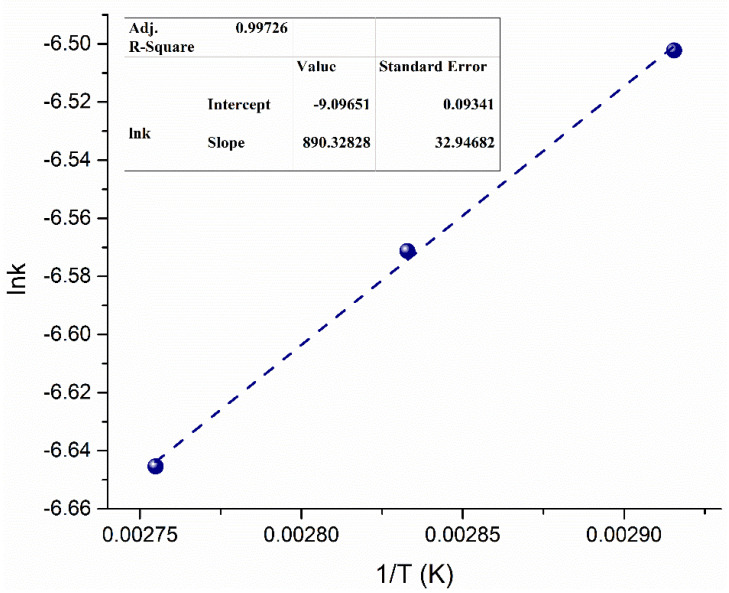
Arrhenius plot for determining *E_A_* required to convert WCO into biodiesel.

To further investigate the kinetics of the vapor–liquid system, the activation energy (*E_A_*) was determined using the Arrhenius equation as a function of the effect of temperature on the rate of reaction [28,41]. Numerous studies determined the *E_A_* of processes using the Arrhenius equation [27,45]. The relationship of k as a function of pre-exponential factor is used to calculate *E_A_* using Equation (9) [46], where *A*° is the pre-exponential factor and *R* is the gas constant (8.314 J·mol^−1^·K^−1^) **(**Figure 8).

(9) $$\ln k=-\frac{E_{A}}{RT}+\ln\;A^{{}^{\circ}}.$$

The *E_A_* of the 7% Sr/ZrO_2_-based biodiesel production process using microbubble technology was estimated as 7.4 kJ·mol^−1^. The achieved *E_A_* is lower than that of other biodiesel processes with different catalysts (Table 4). The low *E_A_* also implies that 7% Sr/ZrO_2_ in the system facilitated the vapor–liquid system and enhanced the overall reaction rate. Furthermore, the low *E_A_* indicates that less energy was needed for the reactant to pass the activation barrier.

Furthermore, 7% Sr/ZrO_2_ has a high surface area and microbubbles have high surface energy, which collectively facilitated the rate of reaction and reduced the *E_A_* of the system. Moreover, MeOH was injected in the form of vapor, indicating that the latent heat of MeOH was also available in the form of free energy, making the nature of the reaction more exergonic. The high reaction rate and reduced *E_A_* demonstrate the potential for implementation of both the heterogeneous catalyst and the vapor–liquid system on a commercial level.

**Table 4:** The comparison of *E_A_* different biodiesel processes.

Feedstock	Type of Transesterification	Catalyst	Activation Energy (kJ.mol^−1^)	Reference
Waste cooking oil	Ultra-Sonication	Calcium diglyceroxide	119.23	[34]
Waste cooking oil	Supercritical method	No catalyst	50.5	[47]
Waste cooking oil	Microwave technology	Calcium diglyceroxide	26.56	[48]
Waste cooking oil	Conventional method	CaO/SiO_2_	66.27	[49]
Waste cooking oil	Conventional method	Cs_2.5_H_0.5_PW_12_O_40_	36	[50]
Stearic acid	Conventional method	ZrO_2_/SiO_2_	47	[51]
Rapeseed oil	Solvent-free method	Sulfated zirconia	22.5	[52]
Levulinic acid	Conventional method	SO_4_^2^^−^/ZrO_2_	14.61	[53]
Oleic acid	Microbubble process	H_2_SO_4_	26.37	[27]
Chicken fat oil	Microbubble process	PTSA	24.9	[28]
Waste cooking oil	Microbubble process	7% Sr/ZrO_2_	7.4	This study

### 3.4. Reusability and Reactivation of the Sr/ZrO_2_

**Figure 9:**
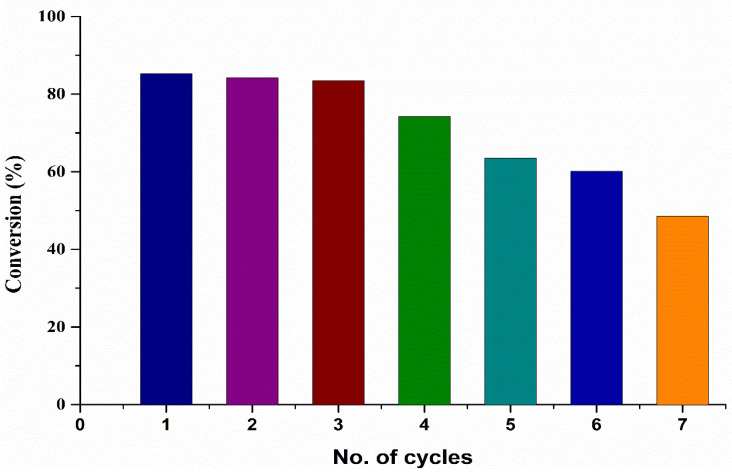
Reusability of the catalyst in the current microbubble technology.

The reusability of the catalyst was evaluated under the optimized conditions obtained in the current study. The 7% Sr/ZrO_2_ catalyst was evaluated for five cycles, with the conversion dropping after each cycle, as shown in Figure 9. After each cycle, the catalyst was centrifuged and washed with MeOH and acetone before reintroducing it into a new cycle. The results show that, after the third cycle, the conversion decreased by less than 10%, whereas, after the seventh cycle, the conversion reached 48%. A possible reason for this behavior is the leaching of Sr ions into the reaction medium, reducing the catalyst activity [54,55]. However, the catalyst can be regenerated after four cycles by loading a certain amount of Sr ions, followed by calcination of the catalyst. Hence, the catalyst can be used for up to four cycles in the current system, after which catalyst reactivation is required.

## 4. Conclusions

A ZrO_2_-based bifunctional heterogeneous catalyst was successfully prepared using strontium nitrate. The physicochemical properties of the catalyst enabled the interaction between ZrO_2_ and strontium nitrate. Moreover, the bifunctional heterogeneous catalyst improved catalytic activity when combined with microbubble technology. The results achieved 85% conversion in 20 min, which is higher than previously reported bifunctional catalysts. The activation energy of the current process was 7.4 kJ·mol^−1^, highlighting the effect of the catalyst on increasing the process efficiency. The catalyst also showed substantial chemical and thermal stability, as it could be reused at least four times without losing biodiesel production activity. The current study provides sufficient evidence for the presence of bifunctional metallic catalysts on the interface of microbubbles in the form of biodiesel processing with a high reaction rate and low activation energy. This study again supports that the use of microbubble technology is a viable alternative for the production of low-cost biodiesel for sustainable energy production.

## Figures and Tables

**Table 1:** WCO properties and its oil composition.

Parameters	Units	Values
Viscosity	mPa·s	31.16
Density	kg·m^−3^	919
FFA	%	9
**Oil composition**		
Linoleic acid	wt.%	9
Linolenic acid	wt.%	62
Palmitic acid	wt.%	12
Lignoceric acid	wt.%	17

**Table 2:** Structural properties of catalyst.

Catalyst	Surface Area (m^2^/g)	BJH Area (m^2^/g)	BJH Volume (cm^3^/g)	BET Pore Diameter (nm)	BJH Pore Diameter (nm)
7% Sr/ZrO_2_	119.80	117.34	0.2154	8.97	8.21

## Data Availability

The data presented in this study are available on request from the corresponding author. The data are not publicly available due to confidentiality of work.
